# The influence of BCL2, BAX, and ABCB1 gene expression on prognosis of adult de novo acute myeloid leukemia with normal karyotype patients

**DOI:** 10.2478/raon-2023-0017

**Published:** 2023-04-20

**Authors:** Zlatko Pravdic, Nada Suvajdzic Vukovic, Vladimir Gasic, Irena Marjanovic, Teodora Karan-Djurasevic, Sonja Pavlovic, Natasa Tosic

**Affiliations:** Clinic of Hematology, Clinical Center of Serbia, Belgrade, Serbia; School of Medicine, University of Belgrade, Serbia; Institute of Molecular Genetics and Genetic Engineering, University of Belgrade, Serbia

**Keywords:** acute myeloid leukemia with normal karyotype, *BCL2*, *BAX*, BCL2/BAX ratio, *ABCB1*, prognosis

## Abstract

**Background:**

Deregulation of the apoptotic process underlies the pathogenesis of many cancers, including leukemia, but is also very important for the success of chemotherapy treatment. Therefore, the gene expression profile of main apoptotic factors, such as anti-apoptotic *BCL2* (B-cell lymphoma protein 2) and pro-apoptotic *BAX* (BCL2-associated X), as well as genes involved in the multi-drug resistance (*ABCB1*), could have significant impact on the prognosis and could be used as targets for specific therapy.

**Patients and methods:**

We analyzed the expression of *BCL2*, *BAX,* and *ABCB1* in bone-marrow samples collected at diagnosis from 51 adult patients with acute myeloid leukemia with normal karyotype (AML-NK) using real-time polymerase chain reaction method, and examined their prognostic potential.

**Results:**

Increased expression of *BCL2* (*BCL2*^+^) was associated with the presence of chemoresistance (p = 0.024), while patients with low *BAX* expression were more prone to relapse (p = 0.047). Analysis of the combined effect of *BCL2* and *BAX* expression showed that 87% of patients with *BAX/BCL2*^low^ status were resistant to therapy (p = 0.044). High expression of *ABCB1* was associated with *BCL2*^+^ status (p < 0.001), and with absence *FLT3-ITD* mutations (p = 0.019).

**Conclusions:**

The present analysis of *BCL2*, *BAX,* and *ABCB1* gene expression profiles is the first study focusing solely on AML-NK patients. Preliminary results showed that patients with high *BCL2* expression are likely to experience resistance to chemotherapy, and may benefit from specific anti-BCL2 treatment. Further investigations conducted on a larger number of patients could elucidate actual prognostic significance of these genes in AML-NK patients.

## Introduction

Acute myeloid leukemia (AML) is a malignant hematological disease that occurs as a result of differentiation arrest, uncontrolled proliferation and diminished apoptosis of myeloid progenitor cells. It is the most common acute leukemia in adults, accounting for about 80% of all cases.^1^ Despite remarkable progress in uncovering the molecular-genetic changes underlying the pathogenesis of AML, a little has changed in the initial treatment of patients which is still based on the classification of patients into risk groups according to pretreatment karyotype analysis.^2^ The largest karyotype based risk group is AML with normal karyotype (AML-NK), representing almost 50% of *de novo* adult AML cases. AML-NK is a highly heterogeneous group with respect to genetic abnormalities and clinical outcome of the patients, but the whole group is still stratified into intermediate risk group. Some of molecular markers such as mutations in fms-related tyrosine kinase-3 (*FLT3*), nucleophosmin (*NPM1)*, CCAAT/enhancer binding protein alpha (*CEBPA*) and runt-related transcription factor 1 (RUNX1) gene have made an impact on prognosis of AML-NK patients, and have already been included into the revised World Health Organization (WHO) classification of myeloid neoplasms and acute leukemia, and European LeukemiaNet (ELN).^2,3^ However, there is a constant need for the introduction of new molecular markers that have significant impact on the prognosis of patients.

Since diminished apoptosis is one of the hallmark traits of leukemic cells, research focused on the analysis of the expression profile of the main participants in the apoptotic process can be of great importance in the detection of new prognostic-relevant molecular markers of AML. Also, since the mechanism of action of cytotoxic drugs used in the treatment of AML involves activation of apoptotic process, the expression pattern of apoptotic factors could have an impact on the occurrence of resistance. Namely, multi drug resistance (MDR) is a main clinical obstacle to successful cancer treatment. Resistance to chemotherapy treatment in AML is still a major cause for initial treatment failure and relapse of the disease, and it is caused by multifactorial mechanisms involving genetic factors.^4,5^ Two main mechanisms of MDR are: pump (transport) resistance, associated with increased expression of proteins involved in drug efflux, and non-pump (apoptotic) resistance, associated with increased activity of anti-apoptotic system.^6^ Gene expression of the ATP-binding cassette (ABC) superfamily of membrane transporters (*ABCB1* formerly known as *MDR1* gene) is a marker of pump-resistance, while markers of non-pump resistance are levels of expression of *BCL2* and *BAX* genes.^7,8,9^ These markers could be considered as pharmacotranscriptomics markers and their analysis in AML patients could be a basis for the assessment of their role in tumor resistance to several groups of drugs. The inhibitors of these markers might effectively reverse MDR in AML patients.^10^

The process of apoptosis is under the control of two distinct but interconnected pathways, intrinsic and extrinsic. Activation of intrinsic pathway is under the control of the BCL family of proteins. B-cell lymphoma 2 (BCL2) family of proteins include both pro-apoptotic (BAX, BAK) and anti-apoptotic members (BCL2, BCL-XL, MCL1).^11^ BCL2 is the best-known member of BCL2-familly, with an anti-apoptotic function. Its high expression has been reported throughout the evolution of AML, at presentation, relapse and also during treatment resistance. Moreover, increased BCL2 correlated with failure to achieve complete remission (CR) and with shorter overall survival (OS) of AML patients, making it an important therapeutic target.^12,13^ Indeed, these findings led to the design of potent and selective BCL2-inhibitor, venetoclax. This modern BCL2-inhibitor is used in combination with classical therapy improving outcome in patients that are ineligible for intensive chemotherapy.^14^

BCL2-associated X (BAX) is a pro-apoptotic protein, transcriptionally activated by the tumor suppressor p53. BAX is essential in the final stages of apoptotic process and its activation leads to release of cytochrome c from mitochondria and direct cell death.^15^ Some studies found that high expression of BAX is good prognostic marker in AML, while others failed to prove its prognostic significance.^16,17,18,19,20^

Because of the existing inconsistencies in assessment of the individual impact of BCL2 and BAX expression level on AML prognosis, researchers have resorted to BAX/BCL2 ratio analysis.^9,19^ Namely, BAX and BCL2 regulate apoptotic process by binding to each other and thus forming heterodimers. The BAX/BCL2 ratio determines the cell fate after apoptotic stimuli has been received.

The overexpression of *ABCB1* gene is considered to be independent factor for the occurrence of multi-drug resistance in AML. *ABCB1* gene is located at chromosome 7q21.31, and it encodes 120 kb permeability glycoprotein (P-gp), a member of ATP-binding cassette (ABC) superfamily of transporter proteins, also called adenosine triphosphate binding cassette transporter B1 (ABCB1). P-gp is an efflux pump, transporting toxic substances out of the cell.^21,22^ By decreasing intracellular concentrations of drugs P-gp confers resistance to a large number of therapeutics used in clinical oncology. Also, P-gp has a drug-independent role in AML causing the inhibition of apoptosis in AML blast cells via modulation of a sphingomyelin-ceramide pathway.^23^

In order to show how the expression level of main apoptotic factors, such as *BCL2* and *BAX*, can influence the occurrence of resistance, and whether their influence is independent from the impact of multi drug resistance (*ABCB1*) gene expression level, in this study we investigated the expression pattern of these genes, and examined the possibility of their mutual influence on the prognosis in AML-NK patients. In doing so, the expression level of these genes was analyzed in the context of other already established prognostic molecular markers. In this way, we aimed to determine how the expression pattern of these genes can be used for a more precise stratification of AML-NK patients into risk groups.

## Patients and methods

### Patients and therapy protocol

Bone marrow (BM) samples from the 51 newly diagnosed AML-NK patients (25 females, 26 males; median age 51 years, range 23–62 years) were collected at Clinic of Hematology, Clinical Center of Serbia. Research was conducted in accordance with the ethical standards of the World Medical Association's Declaration of Helsinki. The study was approved by the Ethics Committee of the Clinical Center of Serbia (No. 110/11), and written informed consent was obtained for all patients.

Diagnostic procedures comprised cytomorphology, cytogenetics, and immunophenotyping of BM. Morphologic diagnosis was made according to the French-American-British (FAB) classification.^24^ Conventional G-band karyotyping was employed for cytogenetic analysis.^25^ Immunophenotyping by flow cytometry (FACS Calibur, BD Biosciences, USA) was carried out systematically in the whole group of patients according to standard protocols based on European LeukemiaNet (ELN), Work Package 10 (WP10) criteria.^26^

All patients received induction and consolidation chemotherapy with daunorubicin and cytarabine according to the protocol 3 + 7, followed by three consolidation cycles of high/intermediate doses of cytarabine.^2^ Patients aged ≤ 55 years underwent allogeneic stem cell transplantation (SCT), in total 15 (25.42%) patients. Definitions of CR, overall survival (OS), disease free survival (DFS) and resistance were established by proposed criteria.^27^

### Gene expression and mutational analyses

Bone marrow mononuclear cells (BMMCs) from AML-NK patients and from 14 healthy controls (BM donors, 8 males and 6 females, median age 31 years), were purified on *Ficoll-Paque*^TM^ Plus (GE Healthcare, Buckinghamshire, UK) density gradient, suspended in TRI Reagent (Ambion, Thermo Fisher Scientific, Waltham, MA, USA) and total RNA was extracted according to manufacturer's instructions. In brief, mononuclear cells where first homogenized in TRI Reagent, and then separation phase was initiated by adding chloroform and subsequent certification. Total RNA was precipitated from the aqueous phase using isopropyl alcohol, pelleted and washed in 70%–75% ethanol. One microgram of total RNA was used for the cDNA synthesis using RevertAid Reverse Transcriptase (Invitrogen, Thermo Fisher Scientific, Waltham, MA, USA). Real time-PCR was performed on 7900HT Fast Real-Time PCR System (Applied Biosystems). For expression analysis of *ABCB1 SYBR®*Green chemistry was used and PCR reaction (10 μl) consisted of 1μl of cDNA (50 ng RNA equivalent) *SYBR™* Green PCR Master Mix (Applied Biosystems, Foster City, CA, USA), primers in final concentration 400 nM (*ABCB1* forward 5′-GTC TAC AGT TCG TAA TGC TGA CGT and *ABCB1* reverse 5′-TGT GAT CCA CGG ACA CTC CTA C). In the case of *BCL2* and *BAX*, expression analysis was done as previously described.^28^ For all target genes *GAPDH* gene was used as endogenous control, and all reactions were run in duplicate. Relative quantification analysis was performed using comparative ddCt method, using healthy controls as calibrator.^29,30^

Detection of *FLT3-ITD* and *NPM1* mutations were analyzed as previously described.^31,32^

### Statistical analysis

Data are presented as medians with range, means ± SD, or as absolute numbers with percentages. Differences in continuous variables were analyzed using Mann-Whitney *U* test for distribution between 2 groups. Analyses of frequencies were performed using Fisher exact test. Survival probabilities were estimated by the Kaplan-Meier method, and differences in survival distributions were evaluated using the LogRank test.

The statistical analyses were performed using the SPSS computer software 21.0 (IBM). For all analyses, the P values were 2-tailed, and *P* < 0.05 was considered statistically significant.

## Results

In the stud y, we analyzed the expression of *BCL2*, *BAX* and *ABCB1* gene, in a cohort of 51 newly diagnosed patients with AML-NK. Clinical and biological characteristics of the patients are shown in Table 1.

**TABLE 1.** Clinical characteristics for *de novo* acute myeloid leukemia with normal karyotype (AML-NK) patients stratified by the level of *BCL2*, *BAX* gene expression and *BAX*/*BCL2* ratio

**Parameter**	**BCL2**				***BAX***			***BAX/BCL2***		

	**Total n=51**	***BCL2+* n=25**	***BCL2−* n=26**	**P**	***BAX+* n=25**	***BAX−* n=26**	**P**	***BAX/BCL2^high^* n=25**	***BAX/BCL2*^low^ n=26**	**P**
**Sex**										
Male (%)	26 (51)	10 (38)	16 (62)	0.165	16 (62)	10 (38)	0.095	16 (62)	10 (38)	0.095
Female (%)	25 (49)	15 (60)	10 (40)		9 (36)	16 (64)		9 (36)	16 (64)	
Age^*^ (years)	51 (23–62)	53 (27–62)	49 (23–62)	0.137	49 (27–62)	52.5 (23–62)	0.597	48 (23–62)	54 (27–62)	0.111
WBC count^*^ (×10^9^/L)	22 (1–349)	7 (1–184)	22.5 (2–349)	0.159	22 (1–184)	22 (0.2–349)	0.808	29 (1–349)	6 (1–184)	**0.041**
HB^*^ (g/L)	99 (66–131)	103 (82–131)	98 (66–128)		97 (66–131)	105 (78–128)		98 (66–128)	103 (82–131)	
> 80 (g/L)	45 (88)	25 (56)	20 (44)	**0.023**	22 (49)	23 (51)	1.000	19 (53)	26 (47)	
< 80 (g/L)	6 (12)	0	6 (100)		3 (50)	3 (50)		0	6 (100)	**0.010**
Plts^*^ (×10^9^/L)	55 (8–422)	60 (8–422)	53.5 (8–169)	0.528	55 (17–169)	54.5 (8–422)	0.497	52 (8–169)	60 (8–422)	0.685
**LDH** ^*^(U/L)	321 (1–2904)	175 (153–1992)	590.5 (1–2904)	**0.010**	386 (153–1992)	306.5 (1–2904)	0.816	605 (1–2904)	75 (153–1992)	**0.002**
PB blast^*^ (%)	14 (0–98)	15 (0–98)	11 (0–97)	0.737	11 (0–92)	16 (0–98)	0.623	11 (0–97)	15 (0–98)	0.865
BM blasts^*^ (%)	62 (30–97)	57 (30–90)	66.5 (32–90)	0.531	63 (30–97)	61 (31–90)	0.756	70 (30–90)	57 (31–97)	0.341
**CD34** (%)				0.095			0.404			**0.050**
present	24 (47)	15 (63)	9 (38)		10 (42)	14 (58)		8 (33)	16 (67)	
absent	27 (53)	10 (37)	17 (363)		15 (56)	12 (44)		17 (63)	10 (37)	
**FAB** (%)				**0.006**			0.239			**0.002**
M0	4 (8)	4 (100)	0		1 (25)	3 (75)		0	4 (100)	
M1	5 (10)	4 (80)	1 (20)		3 (60)	2 (40)		1 (20)	4 (80)	
M2	18 (35)	10 (56)	8 (44)		7 (39)	11 (62)		6 (33)	12 (67)	
M4	17 (33)	3 (18)	14 (82)		8 (47)	9 (53)		14 (82)	3 (18)	
M5	7 (14)	4 (57)	3 (43)		6 (86)	1 (14)		4 (57)	3 (43)	
**CR** (%)				0.404			0.264			0.577
success	28(55)	12 (43)	16 (57)		16 (57)	12 (43)		15 (54)	13 (46)	
failure	23(45)	13 (57)	10 (43)		9 (39)	14 (61)		10 (43)	13 (57)	
**Resistance** (%)				**0.024**			0.703			**0.044**
yes	8 (16)	7 (88)	1 (12)		3 (38)	5 (62)		1 (13)	7 (87)	
no	43 (84)	18 (42)	25 (58)		22 (51)	21 (49)		24 (56)	19 (44)	
**Relapse** (%)				1.000			0.047			0.137
yes	17 (61)	7 (41)	10 (59)		7 (41)	10 (59)		7 (41)	10 (59)	
no	11 (39)	5 (45)	6 (55)		9 (82)	2 (18)		8 (73)	3 (27)	
***FLT3-ITD* mutations** (%)				0.324			1.000			0.199
present	12 (24)	4 (33)	8 (67)		6 (50)	6(50)		8 (67)	4 (33)	
absent	39 (76)	21 (54)	18 (46)		19 (49)	20 (51)		17 (44)	22 (56)	
***NPM1* mutations** (%)				0.237			0.144			0.144
present	17 (33)	6 (35)	11 (65)		11 (65)	6 (35)		11 (65)	6 (35)	
absent	34 (67)	19 (56)	15 (44)		14 (41)	20 (59)		14 (41)	20 (59)	

BM = bone marrow; CR = complete remission; HB = hemoglobin; FAB = French-American-British classification; PB = peripheral blood; Plts = platelets; WBC = white blood cell count

* median (range)

### BCL2 expression

Median expression level of *BCL2* in cohort of 51 AML-NK patients at diagnosis was 1.22 (range 0.13–8.97), which was not significantly different compared to healthy controls (median 1.00, range 0.21–1.59) (P = 0.148). When *BCL2* median expression level detected among AML-NK patients (1.22) was applied as a cut-off value, 49% of patients exhibited high *BCL2* expression, and were marked as *BCL2*^+^ (Table 1).

Examining the association of BCL2 expression level with clinical characteristics of the patients, we have found that BCL2^+^ patients had lower LDH levels (P = 0.010) and higher hemoglobin level (P = 0.023) (Table 1.). Also, BCL2^+^ patients primarily belonged to the M0/M1 FAB group of patients (P = 0.006). The presence of *BCL2*^+^ status was not associated with mutations in *FLT3-ITD* and *NPM1* gene (P = 0.324 and P = 0.237, respectively).

When we analyzed the prognostic impact of high *BCL2* expression in our cohort of AML-NK patients we have found that *BCL2*^+^ status was associated with the presence of resistant disease, since 88% of resistant patient had elevated *BCL2* expression (P = 0.024). The CR rate among our group of patients was 55%. Among *BCL2*^+^-positive patients CR rate was lower (48%), but this was not significantly different compared to *BCL2*^−^ group (62%) (P = 0.404). Survival analysis indicated that *BCL2*^+^ patients had longer duration of CR compared to *BCL2*^−^ patients (11 months *vs*. 9.3 months), but this difference showed no statistical significance after survival analysis was performed (LogRank = 0.46, P = 0.831). A similar result was obtained when analyzing the impact of *BLC2* status on OS (*BCL2*^+^, 6 months *vs.*.*BCL2*^−^, 8 months; LogRank = 2.030, P = 0.154).

### BAX expression

Median expression level of pro-apoptotic *BAX* gene in our cohort of AML-NK patients was 0.92 (range 0.27–2.64), which was not significantly different compared to healthy controls (median 1.09, range 0.41–1.55) (P = 0.704). Based on the *BAX* median expression level the patients were divided into *BAX*^+^ and *BAX*^−^ group. There were no significant associations between *BAX* expression level and clinical and molecular characteristics of the patients.

Analysis of the potential prognostic impact of *BAX* status showed that *BAX*^−^ patients had lower CR rate compared to *BAX*^+^ group, but without statistical significance (P = 0.264) (Table 1). The negative impact of low *BAX* expression was also reflected in the fact that *BAX*^−^ patients were more prone to relapse (P = 0.047). Also, *BAX*^−^ patients had lower DFS (*BAX*^−^, 8 months *vs.*. *BAX*^+^, 11 months; LogRank = 0.020, P = 0.889), and lower OS (*BAX*^−^, 5 months *vs.*. *BAX*^+^, 7 months; LogRank = 0.020, P = 0.888) but it was not statistically significant.

### Combined BCL2 and BAX expression (BAX/BCL2 ratio)

The possible cumulative effect of both *BCL2* and *BAX* gene expression level was also analyzed using *BAX/BCL2* ratio. In AML-NK group median *BAX/BCL2* ratio was 0.62 (range 0.11–7.77), while in healthy samples it was 0.91 (range 0.59–3.69). We haven’t found significant difference between *BAX/BCL2* values among patient and healthy control group (P = 0.185). When median *BAX/BCL2* value detected in AML-NK patients (0.62) was applied as a cut-off value for discriminating *BAX/BCL2*^high^ and *BAX/BCL2*^low^ group, 49% of patients had *BAX/BCL2*^high^ status.

Regarding the clinical characteristics of the patients, *BAX/BCL2*^high^ status was associated with higher number of WBC (P = 0.041), hemoglobin levels lower than 80 g/L (P = 0.010), higher LDH level (P = 0.002), with M4 FAB group (P = 0.002), and with absence of CD34 (P = 0.050). *BAX/BCL2*^high^ status was not significantly associated with mutations in *FLT3-ITD* and *NPM1* gene (P = 0.199 and P = 0.144) (Table 1).

The prognostic significance of *BAX/BCL2* ratio was evident only in terms of the presence of primary resistance, where *BAX/BCL2*^low^ status patients were in 87% resistant to therapy (P = 0.044). Survival analysis didn’t show any significant difference in DFS and OS duration between *BAX/BCL2*^high^ and *BAX/BCL2*^low^ groups of patients (LogRank = 0.139, P = 0.710 and LogRank = −0.004, P = 0.951, respectively).

### ABCB1 expression

In our cohort of AML-NK patients median expression level of *ABCB1* gene was significantly lower compared to healthy controls (0.16, range 0.00–13.74 *vs*. 1.02, range 0.29–5.27, respectively) (P = 0.025). According to median *ABCB1* expression level, we have divided patients into *ABCB1*^+^ (25 patients) and *ABCB1*^−^ group (26 patients). We have found that *ABCB1*^+^ patients had lower LDH levels (P = 0.028), and were predominantly found in M0/M1 FAB group (P<0.001). Furthermore, *ABCB1*^+^ status was associated with the absence of *FLT3-ITD* (P = 0.019), as well as, with the presence of CD34 antigen (P = 0.050) (Table 2).

**TABLE 2.** Clinical characteristics for *de novo* acute myeloid leukemia with normal karyotype (AML-NK) patients stratified by the level of *MDR1* gene expression

**Parameter**	**Total n=51**	**MDR1^+^ n=26**	**MDR1^−^ n=25**	**P**
**Sex**				**0.051**
Male (%)	26 (51)	17 (65)	9 (35)	
Female (%)	25 (49)	9 (36)	16 (64)	
Age^*^ (years)	51 (23–62)	53 (23–62)	49 (23–62)	0.396
WBC count^*^ (×10^9^/L)	22 (1–349)	7 (1–184)	26 (0–349)	0.071
Hemoglobin^*^ (g/L)	99 (66–131)	106 (78–124)	96 (66–131)	0.191
> 80 (g/L)	45 (88)	24 (53)	21 (47)	
< 80 (g/L)	6 (12)	1 (17)	5 (83)	
Platelets^*^ (×10^9^/L)	55 (8–422)	42 (8–422)	69.5 (16–169)	0.129
**LDH** ^*^(U/L)	321 (1–2904)	175 (1–2904)	553.5 (175–1992)	**0.028**
PB blast^*^ (%)	14 (0–98)	14 (0–98)	13.5 (0–87)	0.900
BM blasts^*^ (%)	62 (30–97)	57 (30–90)	65 (33–97)	0.565
**CD34** (%)				**0.025**
present	24 (47)	16 (67)	8 (33)	
absent	27 (53)	9 (33)	18 (67)	
**FAB** (%)				**<0.001**
M0	4 (8)	4 (100)	0	
M1	5 (10)	5 (100)	0	
M2	18 (35)	11 (61)	7 (39)	
M4	17 (33)	3 (18)	14 (82)	
M5	7 (14)	2 (29)	5 (71)	
**Complete remission** (%)				0.781
success	28(55)	13 (46)	15 (54)	
failure	23(45)	12 (52)	11 (48)	
**Resistance** (%)				1.000
yes	8 (16)	4 (50)	4 (50)	
no	43 (84)	21 (49)	22 (51)	
**Relapse** (%)				0.460
yes	17 (61)	9 (53)	8 (47)	
no	11 (39)	4 (36)	7 (64)	
***FLT3-ITD* mutations** (%)				**0.019**
present	12 (24)	2 (17)	10 (83)	
absent	39 (76)	23 (59)	16 (41)	
***NPM1* mutations** (%)				0.075
present	17 (33)	5 (29)	12 (71)	
absent	34 (67)	20 (59)	14 (41)	
***BCL2*** ^+^	25 (49)	20 (80)	5 (20)	**<0.001**
***BCL2^−^***	26 (51)	5 (24)	21 (76)	
***BAX/BCL2*** ^high^	25(49)	5 (20)	20 (80)	
***BAX/BCL2*** ^low^	26 (51)	20 (77)	6 (23)	**<0.001**

BM = bone marrow; FAB = French-American-British classification; PB = peripheral blood; WBC = white blood cell count

* median (range)

Interestingly, *ABCB1* status was not associated with the occurrence of resistance (P = 1.000), nor did it affect the CR rate (P = 0.781). Also, expression level of *ABCB1* did not affect DFS and OS duration (LogRank = 0.037, P = 0.848 and LogRank = 0.951, P = 0.329, respectively).

In addition, patients with high expression of *ABCB1* predominantly had high expression of *BCL2*, and therefore were frequently found in *BAX/BCL2*^low^ group (P < 0.001).

When we performed substratification of AML-NK patients based on the presence of *FLT3-ITD* and *NPM1* mutations into 3 risk groups (favorable *NPM1*^+^-11 patients, poor *FLT3-ITD*^+^-12 patients, and intermediate *FLT3-ITD*^−^/*NPM1*^−^ −28 patients), we have found that *ABCB1*^+^ status was predominant in the *FLT3-ITD*^−^/*NPM1*^−^ group, because 71% of *FLT3-ITD*^−^/*NPM1*^−^ patients had high *ABCB1* expression (P = 0.001). Analyzing the potential prognostic significance of *ABCB1* expression in this group of 28 patients, prominent impact was observed only in survival analysis for OS where *ABCB1*^+^ patients had shorter survival of 5 months, compared to *ABCB1*^−^ patients with 10 months (LogRank = 3.447, P = 0.063).

## Discussion

Loss of control in the process of programmed cell death is one of the basic events in the malignant transformation and the development of various types of tumors, including AML. For this reason, many of the participants in apoptosis are recognized as targets for the design and application of therapeutics. Aberrant expression of genes that control apoptosis, like BCL2-familly members, represent a recurrent feature of leukemic cells that can lead to increased cell survival and chemotherapy resistance.^11,33,34^ In this study we analyzed the expression pattern of two BCL2-family member genes, *BCL2* and *BAX*, as well as *BAX*/*BCL2* ratio in order to elucidate their influence on prognosis of AML-NK patients.

We have found that the expression level of *BCL2* among *de novo* AML patients was not different compared to healthy controls, and showed extremely heterogeneous pattern, with wide range of detected values. Similar finding was reported by others.^11,35^ Also, consistent with some other previously published findings, in our study *BCL2*^+^ status was not a predictor for reduced CR rate, and did not influence DFS and OS.^9,36,37,38^ However, in our cohort of patients a statistically significant association between *BCL2*^+^ status and the existence of resistance was shown. We believe that this finding might be important because *BCL2*^+^ patients may benefit from specific anti-BCL2 therapy. Bilbao-Sieyro *et al*.^38^ came to the same conclusion after they reported that increased *BCL2* expression found in CR and relapsed samples (but not in diagnosis samples) was associated with poor DFS and OS.

Furthermore, we have found that high expression of *BCL2* was detected among patients with FAB M0/M1 subtypes. This is not surprising, given the fact that the expression of BCL2 is differentiation stage specific, being at its highest in immature myeloid progenitors, and decreasing at the final stages of differentiation.^35^ This finding is very important when anti-BCL2 therapy (venetoclax) is used in the treatment of AML. Namely, it was shown that AML patients belonging to FAB M4/M5 subtype can exhibit resistance to this specific therapy. It is assumed that the cause of this resistance lies in the lack of therapeutic target i.e. BCL2, since leukemic cells belonging to these AML subtypes originate from more differentiated hematopoietic cells, having lower, or non-existent BCL2 expression.^39,40^

Our study showed significant association between decreased *BAX* expression level and higher relapse rate. This finding is similar with already published data, but it has to be said that the influence of *BAX* expression level on prognosis in AML was predominantly studied through its association with other apoptotic genes, like *BCL2* (*BAX*/*BCL2* ratio).9,17,19,20,41,42 In our study, *BAX*/*BCL2*^low^ ratio was significantly associated with the presence of the resistance. Other studies showed that increased BAX/BCL2 ratio was associated with increased CR rate,^18,41^ while patients with low *BAX*/*BCL2* had shorter OS.^18^ Following the example of Del Poeta *et al*.^41^ we also tried to prove the association of *BAX*/*BCL2*^high^ ratio with *NPM1*^−^/*FLT3-ITD*^+^ mutational status, but without any success since the presence of these mutations were not associated with *BCL2* and *BAX* expression level when analyzed individually.

Of note is that the methodology used in the referenced studies was different ranging from flow-cytometry, western-blot, to RT-PCR and RNA-seq, therefore their results cannot be entirely comparable. Also, the analysis focusing on only two apoptotic factors, *BCL2* and *BAX*, represents only a simplification of the real situation, where other pro- and anti-apoptotic members of the BCL2-familly interact with each other and determine the final fate of the leukemic cell.

When we analyzed expression pattern of *ABCB1* gene known to be involved in the chemoresistance, we observed some similarity with the results obtained through *BCL2* and *BAX* expression analysis. Thus, similar to *BCL2* expression, *ABCB1* expression analysis showed that *ABCB1*^+^ status was preferably found in M0/M1 FAB subgroup of patients. This was not surprising considering the fact that ABCB1 expression is dependent on the differentiation stage, i.e. that the highest ABCB expression was observed among cells with immature immunophenotype.^43^ In line with this was the finding that *ABCB1*^+^ status was associated with CD34^+^ status. This is also due to the fact that CD34 expression is present in pluripotent hematopoietic cells, and it's down-regulated during differentiation process, as it is the case with ABCB1 expression.^44,45,46^

In our study, we observed mutual exclusion between *FLT3-ITD*^+^ and *NPM1*^+^ status, and high expression of *ABCB1*. Similar finding was reported by others, and in the case of *FLT3-ITD*^+^ patients it is assumed to be a consequence of a loss of *ABCB1* expression under increased proliferative activity caused by the presence of a *FLT3-ITD* mutation.^47,48,49,50^

In our study overexpression of *ABCB1* was not associated with occurrence of chemoresistance, CR rate, and the duration of DFS and OS. This can be explained by the presence of age-dependent association between high expression of *ABCB1* and adverse prognosis. Namely, the clinical relevance of *ABCB1* expression is diminished or completely lost among young adult AML patients, and particularly in pediatric AML patients.^45,51,52,53^ In our study median age of AM-NK patients was 51 years, and 61% of older patients (> 51 years) were *ABCB1*^high^. Based on this, we can say that our cohort of patients is represented by younger adults, in whom the association between *ABCB1* expression and chemoresistance/adverse outcome, is not so evident. However, it may be that the analysis of a larger number of patients could provide a more accurate statistical power for significance.

Also, in addition to the expression of *ABCB1*, the resistance in AML can be influenced by other members of the ABC-transporter family, or even by the expression pattern of some other prognostically significant genes.^55,56,57^ Some studies have shown that contribution to the resistance, and to the overall prognosis is defined by co-expression patterns of many different ABC-transporters, and not by their individual influence.^54,55,58^ It is assumed that some of the ABC transporters have overlapping specificity to a range of substrates, and that their co-expression is responsible for chemoresistance. This is particularly evident in the study by Marzac C *et al*.^54^ where resistant disease among AML patients increased from 21% to a 100% depending of number of overexpressed ABC transporter genes (0 to 3).

In conclusion, our study showed that occurrence of resistance was associated with increased expression of *BCL2*, while patients with low *BAX* expression were more prone to relapse. Combined impact of these two genes analyzed through *BAX/BCL2* ratio showed that AML-NK patients with *BAX*/*BCL2*^low^ status were resistant to chemotherapy. Also, in our cohort of patients *ABCB1* expression level was not a predictor of resistant disease, but we have found association between *ABCB1*^+^ status and the absence of *NPM1* and *FLT3-ITD* mutations, molecular markers with an already established prognostic significance in AML-NK. However, we were unable to demonstrate that *ABCB1* expression could contribute to a more accurate risk stratification in these patients.

This is the first study in which the expression of *BCL2*, *BAX*, *BAX*/*BCL2* ratio and *ABCB1* was examined solely in AML-NK group of patients in which the prognostic influence of cytogenetic aberration, either unfavorable or favorable, could be excluded. This cytogenetically homogenous group of patients are extremely heterogeneous regarding their outcome, and that is why it would be of great importance if expression pattern of some of these genes should prove to be significant for prognosis and response to therapy. Since the research on pharmacotranscriptomics markers in AML is deficient, this study which includes the gene expression analysis involved in both mechanisms of multidrug resistance (apoptotic-dependent and efflux pump-dependent) has provided new data on these potential components of algorithm for individualized, personalized treatment of AML patients.
